# Changes in Natural Foxp3^+^Treg but Not Mucosally-Imprinted CD62L^neg^CD38^+^Foxp3^+^Treg in the Circulation of Celiac Disease Patients

**DOI:** 10.1371/journal.pone.0068432

**Published:** 2013-07-12

**Authors:** Marieke A. van Leeuwen, M. Fleur du Pré, Roy L. van Wanrooij, Lilian F. de Ruiter, H. (Rolien) C. Raatgeep, Dicky J. Lindenbergh-Kortleve, Chris J. Mulder, Lissy de Ridder, Johanna C. Escher, Janneke N. Samsom

**Affiliations:** 1 Laboratory of Pediatrics, Division of Gastroenterology and Nutrition, Erasmus Medical Center-Sophia Children’s Hospital, Rotterdam, The Netherlands; 2 Center for Immune Regulation, Institute of Immunology, University of Oslo and Oslo University Hospital-Rikshospitalet, Oslo, Norway; 3 Department of Gastroenterology and Hepatology, VU University Medical Center, Amsterdam, The Netherlands; 4 Department of Pediatric Gastroenterology, Erasmus MC-Sophia Children’s Hospital, Rotterdam, The Netherlands; Tulane University, United States of America

## Abstract

**Background:**

Celiac disease (CD) is an intestinal inflammation driven by gluten-reactive CD4^+^ T cells. Due to lack of selective markers it has not been determined whether defects in inducible regulatory T cell (Treg) differentiation are associated with CD. This is of importance as changes in numbers of induced Treg could be indicative of defects in mucosal tolerance development in CD. Recently, we have shown that, after encounter of retinoic acid during differentiation, circulating gut-imprinted T cells express CD62L^neg^CD38^+^. Using this new phenotype, we now determined whether alterations occur in the frequency of natural CD62L^+^Foxp3^+^ Treg or mucosally-imprinted CD62L^neg^CD38^+^Foxp3^+^ Treg in peripheral blood of CD patients. In particular, we compared pediatric CD, aiming to select for disease at onset, with adult CD.

**Methods:**

Cell surface markers, intracellular Foxp3 and Helios were determined by flow cytometry. Foxp3 expression was also detected by immunohistochemistry in duodenal tissue of CD patients.

**Results:**

In children, the percentages of peripheral blood CD4^+^Foxp3^+^ Treg were comparable between CD patients and healthy age-matched controls. Differentiation between natural and mucosally-imprinted Treg on the basis of CD62L and CD38 did not uncover differences in Foxp3. In adult patients on gluten-free diet and in refractory CD increased percentages of circulating natural CD62L^+^Foxp3^+^ Treg, but normal mucosally-imprinted CD62L^neg^CD38^+^Foxp3^+^ Treg frequencies were observed.

**Conclusions:**

Our data exclude that significant numeric deficiency of mucosally-imprinted or natural Foxp3^+^ Treg explains exuberant effector responses in CD. Changes in natural Foxp3^+^ Treg occur in a subset of adult patients on a gluten-free diet and in refractory CD patients.

## Introduction

Celiac disease (CD) is a chronic inflammatory disease of the small intestine that develops in genetically susceptible individuals in response to the ingestion of gluten from wheat, barley and rye. Inflammatory gluten-specific CD4^+^ T cells that are restricted to HLA-DQ2 or HLA-DQ8 molecules can be isolated from the small intestinal mucosa of CD patients but not from healthy individuals [1], [2]. These inflammatory gluten-specific T cells produce large amounts of interferon-γ [3] and are expected to be key contributors to intestinal tissue damage. Currently, the only treatment for CD is a lifelong gluten-free diet (GFD), resulting in complete remission and recovery of the normal intestinal architecture. However, a severe complication occurs in a small proportion of CD patients who become unresponsive to the GFD and develop refractory celiac disease (RCD). RCD is defined by the identification of malabsorption and persisting duodenal villous atrophy, despite adherence to a GFD and absence of other enteropathies [4], [5]. A subgroup of RCD patients, denoted as type II, have aberrant populations of T cells lacking the surface expression of CD3 rendering these patients at high risk to develop an enteropathy-associated T-cell lymphoma (EATL) [6].

Despite our increasing knowledge of the pathogenesis of CD, it is still unclear why oral tolerance to gluten is so often lost and why the excessive pro-inflammatory T-cell response in CD is not suppressed by a regulatory T-cell response. Several regulatory T cell (Treg) subsets have been described to be important for immune tolerance. On the basis of their origin they can be divided in thymus-derived natural Treg cells and the peripherally induced Treg cells [7]. Both subsets share the transcription factor forkhead box P3 (Foxp3). Natural Treg cells primarily maintain tolerance to self-antigen and prevent auto-immunity [8]. On the basis of murine models it has been postulated that natural Treg cells are dispensable for protein specific oral tolerance [9]. In contrast, induced Tregs that have differentiated from naive T cells in the tolerogenic environment of the gut-draining lymph nodes can mediate protein specific oral tolerance in these models [10], [11], [12], [13], [14]. Currently, it is technically impossible to study gluten-specific Treg in patients. However, changes in peripheral blood Foxp3^+^ Treg cells and lamina propria Foxp3^+^ cells have been reported in CD patients. Most studies describe an increase of Foxp3^+^ cells in CD patients compared to controls either in peripheral blood [15] or in the small intestinal mucosa [16], [17], [18], [19], [20]. However, in other studies, no difference in Foxp3^+^ cells was observed between CD patients and controls [21], [22], [23]. As CD patient populations with different ages and clinical status were investigated and because of the large variability in data we hypothesize that changes in Foxp3^+^ T cells may be transient and possibly related to a particular subset of CD patients.

Earlier studies have not investigated whether the increase of circulating Foxp3^+^ Treg cells occurred in the natural Treg cell or induced Treg cell population. This is of importance as changes in numbers of induced Treg could be indicative of defects in mucosal tolerance development in CD. Previously, there was a lack of cell surface markers to distinguish mucosally-imprinted Treg cells from natural Treg cells. Recently, we have demonstrated that mucosally-imprinted CD4^+^ T cells can be identified by the expression of CD62L^neg^CD38^+^ [24]. In mice, this mucosal CD62L^neg^CD38^+^ T-cell phenotype is efficiently induced during differentiation in the gut-draining lymph node which can be mimicked by differentiation in the presence of retinoic acid (RA) and TGF-β. For human CD4^+^ T cells [24], RA alone is sufficient for CD62L^neg^CD38^+^ induction. In human peripheral blood these mucosally-imprinted CD4^+^CD62L^neg^CD38^+^ T-cells have enriched expression of the gut-homing chemokine receptor C-C chemokine receptor type 9 (CCR9) and β7-integrin whereas expression of the skin-homing marker cutaneous leukocyte-associated antigen is almost absent [24]. Crucially, staining of peripheral blood from CD patients who underwent a challenge revealed that virtually all gluten-specific DQ2 tetramer-positive T cells had the CD62L^neg^CD38^+^ phenotype [24]. With this new set of markers we can now distinguish between the mucosally-imprinted Foxp3^+^ T cells and the non-mucosally-imprinted cells.

In this study, we determined whether alterations in the frequency of circulating mucosally-imprinted CD62L^neg^CD38^+^Foxp3^+^ Treg cells are detected in CD patients. We analyzed the percentage of Foxp3^+^ Tregs in the whole circulating CD4^+^ T cell population and within the mucosally-imprinted CD62L^neg^CD38^+^ population. In view of the variability in the results of previous Foxp3^+^ quantification we focused our analysis on a relatively homogenous patient population of pediatric untreated CD patients aiming to select for disease at onset. For comparison we analyzed adult patients with RCD, adult patients receiving a GFD and compared them with healthy adult controls.

## Methods

### Patients (see Table 1 and 2)

Pediatric patients who underwent an esophagogastroduodenoscopy (EGD) with suspicion of CD were approached for participation in our study at the Erasmus Medical Centre – Sophia Children’ s Hospital, Rotterdam, The Netherlands. After diagnosis, those with biopsy-proven CD and positive auto-antibodies (n = 34) or with a Marsh score 1–2, positive auto-antibodies and response to GFD (n = 2) were included in the patient group (n = 36). Whereas children with a normal intestinal histology and negative auto-antibodies were included in the control group (n = 20). Patients diagnosed with other diseases were excluded from the study. Adult patients with treated CD (i.e. CD patients responding to a GFD) and RCD (i.e. CD patients not responding to a GFD) from the VUMC, Amsterdam, The Netherlands were included in the study. The studies were approved by the medical ethical committee of the Erasmus Medical Centre and all participants or parents of participants gave written informed consent before enrollment.

### Serology

Antibodies (IgA) against tissue transglutaminase (tTG) were determined in serum by enzyme-linked immunosorbent assay using either a commercial assay (Thermo Fisher/Phadia, Freiburg, Germany), according to the manufacturer’s instructions, or an in-house assay based on recombinant human tTG (Diarect AG, Freiburg, Germany) as a substrate. Antibodies (IgA) against endomysium were determined in serum by indirect immunofluorescence analysis (IFA) using either commercial primate esophagus slides (Inova, San Diego, CA), according to the manufacturer’s instructions, or in-house primate esophagus slides according to a previously described procedure [25]. All antibody assays used were validated and subjected to both internal and external quality assessment.

### Flow Cytometry

After erythrocyte lysis, whole blood samples were stained for flow cytometry using monoclonal antibodies against CD3 (HIT3α), CD4 (RPA-T4), CD38 (HIT2), CD62L (DREG-56, all BD-Pharmingen), CD45RA (MEM-56, Invitrogen, Breda, The Netherlands), CCR9 (248621, R&D Systems, Abingdon, UK). Intracellular staining was performed with the Foxp3 staining buffer kit, according to manufacturer’s protocol (eBioscience), followed by Foxp3 (PCH101, EMELCA Bioscience, Bergen op Zoom, The Netherlands), Helios (Biolegend, San Diego, US) and the appropriate isotype controls. Flow cytometric analysis was performed on a FACSCanto™II (BD-Biosciences).

### Immunohistochemistry

Immunohistochemical Foxp3 stainings were performed on paraffin-embedded duodenal biopsies as described previously [26]. Prior to staining the samples were blocked with 10% normal human serum in a solution containing Tris buffer (10 mM), EDTA (5 mM), NaCl (0.15 M), gelatine (0.25%) and Tween-20 (0.05%). Tissue sections were incubated overnight using the primary antibody to Foxp3 (eBioscience) or isotype control mouse IgG_1_. Sections were incubated with VECTASTAIN ABC Elite Kit (Vector Laboratories) and stained with biotinylated horse-anti-mouse (Vector Laboratories) as described previously [26].

### Cytokine Analysis

IL-15 concentrations in plasma were analyzed using an enzyme-linked immunosorbent assay set (BD Biosciences) according to the manufacturer’s instructions.

### Statistics

Linear regression analysis was performed using Prism software (GraphPad, Software Inc) the Kruskal-Wallis one-way analysis of variances. Differences between multiple groups were first evaluated by ANOVA using the Kruskal-Wallis test. Differences between groups were analyzed using the Mann-Whitney *U* test. *P*<0.05 was considered statistically significant.

## Results

### Pediatric Patients

As we hypothesize that changes in numbers of circulating Foxp3^+^ T cells may be transient and possibly different in pediatric and adult CD, we first collected a well-defined cohort of newly diagnosed pediatric patients. Blood was drawn from children aged 1 to 17 years who underwent an EGD with a suspicion of CD that were not on a GFD. After diagnosis, those with biopsy-proven CD and positive auto-antibodies (n = 34) or with a Marsh score 1–2, positive auto-antibodies and response to GFD (n = 2) were included in the patient group (n = 36). Whereas children with a normal intestinal histology and negative auto-antibodies were included in the control group (n = 20). CD patients were diagnosed with Marsh I (2 patients), Marsh IIIA (9 patients), Marsh IIIB (17 patients) or Marsh IIIC (8 patients). Patients who were diagnosed with other diseases were excluded from analyses. There was no difference in age between pediatric CD patients and control patients. Demographic features are summarized in Table 1.

**Table 1:** Demographic features of pediatric celiac disease (CD) and controls.

		Pediatric CD	Controls
Number		36	20
Age in years, mean		5.9	6.1
Male, n (% )		n = 9 (25%)	n = 8 (40%)
Marsh score, n (%)	Marsh 0	n = 0 (0%)	n = 20 (100%)
	Marsh 1	n = 2, (6%)	n = 0 (0%)
	Marsh 2	n = 0 (0%)	n = 0 (0%)
	Marsh 3A	n = 9 (25%)	n = 0 (0%)
	Marsh 3B	n = 17 (47%)	n = 0 (0%)
	Marsh 3C	n = 8 (22%)	n = 0 (0%)

CD, celiac disease.

### No Difference in Numbers of Circulating Treg Cells or Mucosally-imprinted Treg Cells between Pediatric CD Patients and Controls

**Figure 1:**
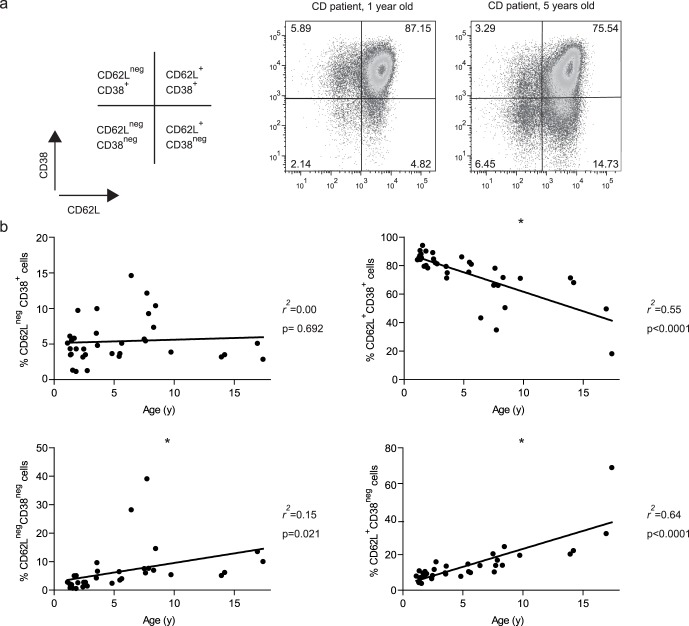
Distribution of CD62L/CD38 subsets within CD4^+^ peripheral blood lymphocytes; changes with age. Peripheral blood from pediatric CD patients was stained for flow cytometric analysis. (**a**) Representative gating strategy for analysis of CD62L/CD38 subsets within the CD4^+^ T cell population. Two representative CD62L/CD38 analyses of peripheral blood CD4^+^ T cells in CD patients aged 1 and 5 years old. (**b**) The percentages of cells in each of the four CD62L/CD38 T-cell subsets were calculated (Kruskal-Wallis test). CD, celiac disease.

**Figure 2:**
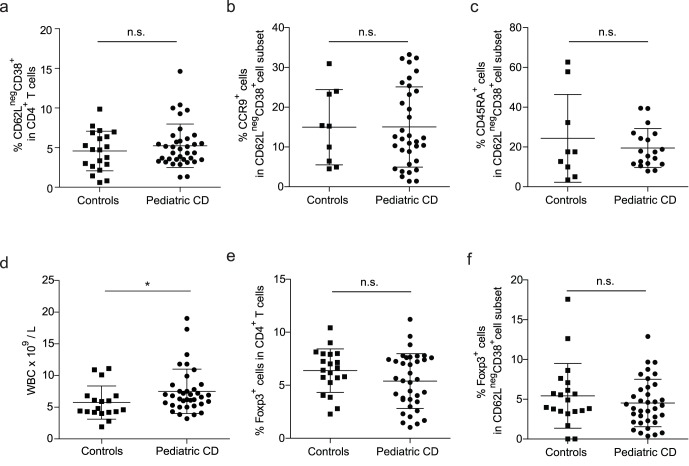
No differences in numbers of circulating Treg cells or mucosally-imprinted Treg cells between pediatric CD patients and controls. (**a**) The percentage of CD62L^neg^CD38^+^ mucosally-imprinted T cells were observed within the peripheral blood CD4^+^ T cell population of CD patients (n = 36) and controls (n = 20). (**b**) The percentage of CCR9^+^ cells within the CD62L^neg^CD38^+^CD4^+^ T cell subset in pediatric CD patients (n = 34) and controls (n = 9). (**c**) Percentages of naive (CD45RA^+^) cells within CD62L^neg^CD38^+^CD4^+^ T cell in pediatric CD patients (n = 19) and controls (n = 9).(**d**) WBC counts per liter peripheral blood for pediatric CD patients (n = 36) and controls (n = 20). (**e**) The frequency of total Foxp3^+^ cells (gated on CD4^+^ lymphocytes) in pediatric CD patients (n = 36) and controls (n = 20). (**f**) The percentage of mucosally-imprinted Foxp3^+^CD62L^neg^CD38^+^ cells in pediatric CD patients (n = 36) and in controls (n = 20). * Statistically significant (*P*<0.05), n.s. not significant (Mann-Whitney *U* test). CD, celiac disease.

Previously, we have established that mucosally-imprinted T cells in peripheral blood can be identified by the expression of CD62L^neg^CD38^+^ [24]. As this is the first cohort of CD patients in which the phenotype of the mucosal the CD62L^neg^CD38^+^ population was extensively analyzed we controlled for several other markers to validate the use of the CD62L^neg^CD38^+^ phenotype in this cohort. It is known that the distribution of lymphocyte subsets in peripheral blood varies with age [27]. Therefore, we first determined whether the distribution of CD4^+^ T cells within the CD62L and CD38 T cell subsets varied with age. Gating of CD4^+^ T cells based on the expression of CD62L and CD38 was performed as shown in Figure 1a. CD4^+^CD62L^+^CD38^+^ T cells gradually decreased from a very high percentage (85.6% ± SD 5.02) in children between 1 and 2 years of age to a much lower and more variable percentage in children over the age of 5 (56.6% ± SD 18.02, Figure 1b). In contrast to the other CD62L/CD38 subsets, the population of CD4^+^CD62L^neg^CD38^+^ mucosally-imprinted T cells was not subjected to age-related changes (Figure 1b). Hence, no age distinction was made for analysis of the pediatric CD patient group for the CD4^+^CD62L^neg^CD38^+^ cells. In addition, no differences were found between pediatric patients and controls in the overall percentage of mucosally-imprinted CD62L^neg^CD38^+^ T cells within the total CD4 population (Figure 2a). The percentages of gut-homing CCR9 positive cells did not differ between patients and controls (Figure 2b). The frequency of naive CD45RA^+^ cells within the mucosal population was also similar in both groups (Figure 2c). It should be noted that the group of CD patients had a significantly higher WBC count in comparison with the control group (Figure 2d). However, all WBC subpopulations were slightly increased and no selective enhancement of CD3^+^ or CD4^+^ cells was seen in patients when compared to controls (data not shown).

To establish whether overall differences in the frequency of Foxp3^+^ cells were detectable we determined the percentage of Foxp3^+^ T cells within the total CD4^+^ T cell population. No difference in the percentage of Foxp3^+^ cells within the total CD4^+^ T cell population was detected between the pediatric CD patient group and the control group (Figure 2e). As only 4% of peripheral blood CD4^+^ T cells have the CD62L^neg^CD38^+^ mucosal phenotype the sensitivity of detection of differences in the mucosally-imprinted Foxp3 population is greatly enhanced. Therefore in our pediatric CD cohort we determined whether these circulating mucosal Tregs were altered in frequency. Despite a clear detectable population of Foxp3^+^ within the CD62L^neg^CD38^+^ population, no difference in the percentage of Foxp3 was detected in the CD62L^neg^CD38^+^ mucosally-imprinted T cell subset upon comparison of pediatric CD with controls (Figure 2f). In addition, no difference was found in the percentage of CCR9^+^ cells within the mucosally-imprinted CD62L^neg^CD38^+^ Foxp3^+^ T cell subset from patients (26.5% ± SD 13.7) when compared to controls (31.0% ± SD 25.4). These data infer that patient and control mucosally-imprinted Tregs should have similar capacity to receive CCR9-mediated CCL25 signals. Also no differences in the percentages of Foxp3 were observed in the other three CD62L and CD38 subsets of pediatric CD patients versus controls (not shown).

Overall, this demonstrates that in a well-defined patient cohort of pediatric CD patients no difference in the frequency of total or mucosally-imprinted Foxp3^+^ T cells can be observed.

### Adult Patients

Having established that significant numeric deficiency of mucosal or systemic Foxp3^+^ T cells cannot explain the exuberant effector response in untreated pediatric CD, we hypothesized that alterations in Foxp3^+^ T cell frequencies may occur in more chronic forms of disease or only in adult patients. As our pediatric cohort contained only patients with active disease before diagnosis and before GFD, we collected peripheral blood of patients with a severe form of CD that is refractory to a GFD (RCD) (n = 14) and adult patients with treated CD (n = 13). Adult patient blood was compared with blood of healthy volunteers (n = 14). Demographic features of the adult patient groups and controls are depicted in Table 2. All treated CD patients were on a GFD for at least 6 months and, as a result of this treatment, their Marsh scores had improved from Marsh 3A–C at the time of diagnosis to a Marsh 0 or 1 and auto-antibody concentrations were negative at the time of blood sampling. Patients who were not responding to a GFD and diagnosed with RCD type I or type II were included in the RCD group. As shown in table 2, the RCD patient group consisted of a heterogeneous population of patients. All RCD patients had an earlier diagnosis of RCD and had a history of treatment with immunomodulatory drugs, including cladribine or ***6***-***thioguanine.*** The latest available Marsh scores of RCD patients varied from Marsh 0 to Marsh 3C. Mucosal healing was seen in RCD patients who received treatment with immunomodulatory drugs, such as cladribine. Three patients with an EATL were also included. No differences in the WBC count were detected between the different patient groups and controls (data not shown).

**Table 2:** Demographic features of adult celiac disease (CD) patients and controls.

		Refractory CD	Treated CD	Healthy controls
Number		14	13	14
Age in years, mean (SD)		60.5 (11.0)	46.1 (14.7)	36.5 (11.5)
Male, n (%)		6 (43%)	6 (55%)	4 (29%)
**Positive antibodies**				
(EmA, tTG), n (%)		1 (7%)*	0 (0%)	0 (0%)
Marsh score, n (%)	Marsh 0	6 (43%)	7 (54%)	not determined
	Marsh 1	3 (21%)	6 (46%)	
	Marsh 2	0 (0%)		
	Marsh 3A	2 (14%)		
	Marsh 3B	2 (14%)		
	Marsh 3C	1 (7%)		
Diagnosis, n (%)	RCD 1	7 (50%)		
	RCD 2	5 (35%)		
	EATL	3 (21%)		
Treatment, n (%)	No	0 (0%)	0 (0%)	14 (100%)
	GFD	13 (93%)	13 (100%)	
	Immunoregulatory drugs	9 (64%)	0 (0%)	
	Unknown	1 (7%)	0 (0%)	

CD, celiac disease; EmA, anti-endomysial antibodies; tTG anti-tissue transglutaminase antibodies; RCD, refractory celiac disease; EATL, enteropathy-associated T cell lymphoma; GFD, gluten free diet. All patients were tested for anti-tissue transglutaminase and anti-endomysial antibodies. * This patient was positive for anti-tissue transglutaminase antibodies.

### Increased Numbers of Circulating Natural Treg Cells in Adult RCD and Treated CD Compared to Controls

**Figure 3:**
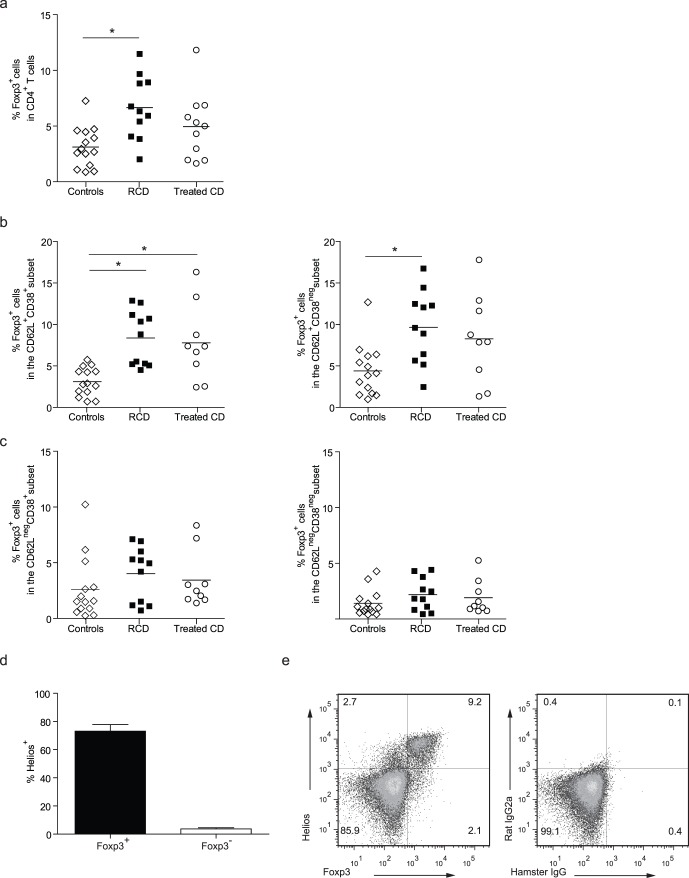
Increased numbers of circulating natural Treg cells in adult RCD and treated CD compared to controls. Peripheral blood was obtained from adult patients with RCD (n = 14), CD patients responding to a GFD (treated CD, n = 13) and healthy controls (n = 14). (**a**) The percentages of Foxp3 cells within the total CD3^+^CD4^+^ T cells and within the different CD4^+^CD62L/CD38 T cells were determined. (**a**) Percentage of Foxp3^+^ cells within the total CD4^+^ T-cell gate (**b**) The percentage of Foxp3^+^ T cells in the CD62L^+^CD38^+^ and CD62L^+^CD38^neg^ subset. (**c**) The percentage of Foxp3^+^ in the mucosally-imprinted CD62L^neg^CD38^+^ T cells or in the CD62L^neg^CD38^neg^ T-cells in patient groups and controls. (**d**) Lymphocytes of 4 RCD patients were gated on CD3^+^CD4^+^ T cells and Foxp3 and Helios positivity were analyzed. The large majority of the Foxp3^+^ cells co-expressed Helios. (**e**) Representative dot-plots of Foxp3 and Helios expression (left panel) and isotype controls (right panel) are shown. Data analyzed Mann-Whitney *U* test. CD, celiac disease; RCD, refractory celiac disease.

Strikingly, a higher percentage of circulating Foxp3^+^ T cells was observed in the total CD4^+^ T cell population in patients with RCD in comparison with healthy controls (Figure 3a). Analysis of the different CD62L/CD38 T cell subsets in RCD patients revealed that this increase in circulating CD4^+^Foxp3^+^ cells was explained by higher proportions of Foxp3^+^ cells in both CD62L^+^ cell subsets suggestive of a more naive phenotype (Figure 3b). Moreover, no changes were observed the mucosally-imprinted (CD62L^neg^CD38^+^) or in the other memory T cell containing CD62L^neg^ T cell subset (Figure 3c). In treated CD patients, the percentage of Foxp3^+^ cells was significantly increased in the CD62L^+^CD38^+^ subset, a subset which contains about 70% CD45RA^+^ cells [24]. Together, these data infer that the increase in Foxp3 in RCD and treated CD patients can be attributed to changes in the natural Treg cell population but not to changes in percentages of the mucosally-imprinted Foxp3^+^CD62L^neg^CD38^+^ Treg cells.

Recent studies have reported that expression of the transcription factor Helios is heterogeneous in Foxp3^+^ Treg [28], [29]. Helios is a marker for T cell activation and in Foxp3^+^ cells Helios expression may select for more proliferative cells that secrete low levels of effector cytokines [30], [31]. Therefore, we examined Foxp3 and Helios expression in CD3^+^CD4^+^ T cells in peripheral blood of 4 RCD patients. The Foxp3^+^ cells virtually all co-expressed Helios (Figure 3d). Representative flow cytometric dot-plots of Foxp3 and Helios expression and the isotype negative controls are shown in Figure 3e.

These data established that numeric increases in systemic Helios^+^ Foxp3^+^ T cells, but not mucosally-imprinted Foxp3 are associated with more chronic forms of disease in adult patients.

Similar to our findings with pediatric CD patients, we did not observe any significant differences in the percentage of total CD4^+^ T cells nor in the distribution of the four CD62L and CD38 T cell subsets in any of the patients groups (Figure S1a). As such, the frequency of mucosally-imprinted CD62L^neg^CD38^+^ T cells was not increased in CD patients with active small-intestinal inflammation (data not shown). We found no differences in the percentage of CD45RA^+^ cells within the CD4^+^ T cell population between patients and controls (Figure S1b). Neither did we observe differences in the expression of CCR9 (Figure S1c and S1d). As seen in pediatric patients, no difference was found in the percentage of CCR9^+^ cells within the mucosally-imprinted CD62L^neg^CD38^+^ Foxp3^+^ T cell subset from adult RCD patients (20.6% ± SD 14.1), treated CD patients (14.3% ± SD 6.9) when compared to controls (15.2% ± SD 5.4).

### Increased Numbers of Foxp3^+^ Cells in the Lamina Propria of Pediatric and Adult CD Patients

Maintenance of intestinal homeostasis requires that Foxp3^+^ Tregs are recruited to the small intestinal tissue. To examine a possible defect in Foxp3^+^ T cell recruitment we performed immunohistochemical stainings to detect Foxp3^+^ cells in duodenal biopsies from pediatric CD patients, adult CD patients, RCD patients and controls. Correlation with disease score revealed an increase in numbers of Foxp3^+^ cells which was already detectable in patients with lower Marsh scores 1–2 (Figure S2). Similar to the pediatric CD patient group, we found increased numbers of Foxp3^+^ cells in the lamina propria of adult CD patients compared to controls (Figure S3). In the treated CD group the numbers of Foxp3^+^ cells were variable between patients. However, in the small number of RCD patients that was evaluated no increased Foxp3 positivity was seen. These data show that in our cohorts of pediatric and adult CD patients increased numbers of Foxp3^+^ cells are detected locally in the inflamed intestinal mucosa and infer that recruitment of Foxp3^+^ cells to the intestine is intact.

### No Increase of IL-15 Plasma Levels in CD Patients

**Figure 4:**
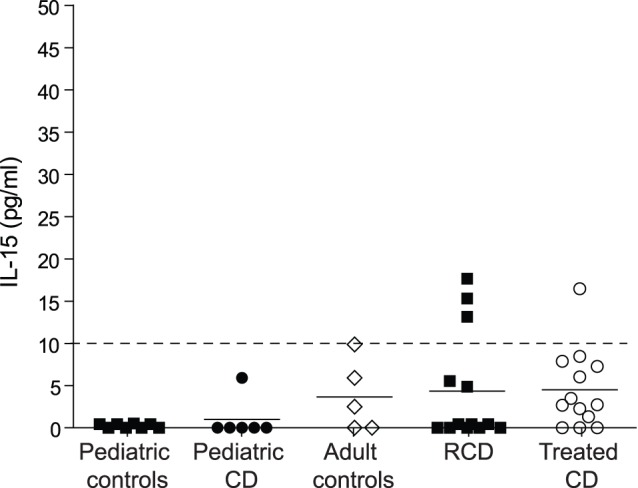
No increase of IL-15 plasma levels in CD patients. The IL-15 concentration in plasma was determined in pediatric controls (n = 8), pediatric CD (n = 6), adult controls (n = 5), adult treated CD (n = 13) and RCD patients (n = 13) by enzyme-linked immunosorbent assay. Dashed line represents the detection limit. CD, celiac disease. Data are representative for three independent measurements.

The cytokine interleukin (IL)-15 has been reported to stimulate Foxp3^+^ Treg cells [32]. In CD patients, IL-15 mRNA is over expressed in the small intestine and increased IL-15 concentrations are detected in serum [33], [34], [35]. Therefore, we investigated whether IL-15 plasma levels were related to increased frequencies of Foxp3^+^ Treg cells in peripheral blood of, adult CD patients. We were able to detect low levels IL-15 in plasma of some CD patients and controls. However, no relation between IL-15 plasma levels and increased frequencies of Foxp3^+^ Treg cells was detected (Figure 4).

## Discussion

Here we show that the frequency of natural and mucosally-imprinted Foxp3^+^ Treg cells is unaltered in the circulation of pediatric CD patients. In contrast, in treated adult CD and RCD patients increased frequencies of natural Foxp3^+^ Treg were detected whereas the mucosally-imprinted CD62L^neg^CD38^+^ subset remained unchanged. As such, systemic changes in natural CD4^+^Foxp3^+^ frequency are not inherent to all CD patients and appear related to a particular phase of disease. This is in contrast to the increased frequency of Foxp3^+^ cells within the lamina propria on histology which was observed in biopsies from both pediatric as well as adult CD patients.

In murine models for oral tolerance to dietary antigen inducible Foxp3^+^ T cells have been shown to differentiate from naive T cells in the mesenteric lymph node under the control of CD103^+^ DC that secrete TGF-β and RA [11]. Recently, we have shown that, in both human and mouse; mucosal imprinting in the mesenteric lymph node leads to a particular T cell phenotype characterized by low levels of CD62L expression and increased levels of CD38. In agreement, DQ2 tetramer staining revealed that gluten-specific T cells appearing in blood of treated celiac disease patients after oral gluten challenge were predominantly CD4^+^CD62L^neg^CD38^+^. As this CD62L^neg^CD38^+^ T cell phenotype is maintained upon entering the circulation [24] it was used in the current study to distinguish mucosally-imprinted T cells within the total CD4^+^ T cell pool. Here we report that no detectable changes are found in the frequency of circulating mucosally-imprinted CD4^+^CD62L^neg^CD38^+^ Foxp3^+^ cells in pediatric or adult CD patients. These data establish that there are no gross defects in mucosal Treg induction in non-treated CD. However, it is not excluded that transient alterations in gluten-specific Foxp3^+^ Treg differentiation occur during the disease process.

Alternatively, the loss of tolerance to gluten in CD could be due to a defect in the effector phase of Treg function within the lamina propria rather than at the level of Foxp3 T-cell differentiation within the mesenteric lymph node. Indeed, multiple studies have provided evidence for such a localized loss of Foxp3 T-cell function. In particular, presence of IL-15 has been reported to abrogate suppression of isolated lamina propria effector T cells by intestinal Foxp3^+^ cells in co-culture [20]. This defective suppression may in part be explained by an IL-15 induced resistance of effector T cells to suppression [34]. Similarly, IL-21 has been suggested to abrogate Foxp3^+^Treg function [36]. Loss of sensitivity to Treg mediated suppression may in consequence allow inflammatory T cells to respond to gluten and to self antigen that is exposed upon tissue damage.

Treg mediated suppression requires sufficient Treg numbers to be present in the inflamed area to counteract the effector T cells. As has been reported in several other studies we observed increased frequencies of Foxp3^+^ cells in tissue biopsies of both pediatric and adult CD patients suggesting that recruitment of Foxp3^+^ T cells to the inflamed tissue is intact or even increased [16], [18], [19], [20], [34]. Unfortunately, immunohistochemistry can not distinguish between natural Tregs and mucosally imprinted Tregs. However, a large proportion of mucosally-imprinted CD4^+^CD62L^neg^CD38^+^ Foxp3^+^ cells from CD patients expressed the chemokine receptor CCR9 which should enable them to respond to CCL25, the chemokine required for small intestinal homing. Also in treated CD patients, numbers of lamina propria Foxp3^+^ cells were increased. We speculate that this may be due to residual inflammation despite a GFD [37]. Contrary to our expectations, in the small number of RCD patients (n = 5) no increased Foxp3 positivity was observed. Further investigation in a larger cohort of patients with defined immunosuppressive treatment is required to establish whether the absence of increased Foxp3^+^ T cell numbers in lamina propria is a general phenomenon associated with RCD.

Changes in circulating Foxp3^+^ T cells in CD patients have been observed in previous studies. However, until now it had not been investigated whether these cells were natural Tregs or induced Tregs. Here we demonstrate that the frequency of CD62L^+^ natural Foxp3^+^ T cells is increased in a subset of adult CD and RCD patients that are treated with a GFD but not in pediatric CD. Interestingly, these Foxp3^+^ cells virtually all expressed Helios. These data infer that changes in the frequency of circulating natural Foxp3^+^ T cells are not inherent to CD but are restricted to a subgroup of CD patients. In first instance, when combining all studies, increased frequencies of circulating Foxp3^+^ cells seem restricted to adult patients [15], [21], [23]. However, this is not observed in all adult cohorts [34]. The variability of these data in the different adult patient cohorts shows that the changes in circulating Foxp3^+^ T cells may be transient and possibly related to a particular stage of inflammation in CD patients. As increases in peripheral Foxp3^+^ Tregs have also been reported for patients with cancer [38], [39], primary Sjögren’s Syndrome and rheumatoid arthritis [40], psoriasis [41] and systemic sclerosis [42], we hypothesize that a non-specific chronic inflammatory mediator can cause this effect. To assess whether IL-15 levels in the circulation were related to increased frequencies of circulating Foxp3^+^ cells we determined IL-15 concentrations in patient plasma. Overall the IL-15 levels did not reveal a possible role of IL-15 in Foxp3 expansion in CD. From this we conclude that circulating CD4^+^Foxp3^+^ T cell numbers are increased in adult patients with RCD and treated CD and hypothesize that this phenomenon may be related to a particular pattern of inflammation involving systemic immune activation.

Overall we conclude that the population of mucosally-imprinted CD62L^neg^CD38^+^ Foxp3^+^ Treg cells has a normal frequency in blood of CD patients suggesting that there are no severe general defects in induction of mucosal Treg cells. Specific defects in gluten-reactive mucosal Tregs can not be excluded but are currently technically impossible to determine. Increased frequencies of Foxp3^+^ natural cells are found in a subgroup of adult CD patients and may be related to systemic inflammation. In the lamina propria of all CD patients Foxp3^+^ T cells are present in the inflammatory lesions but may be inactivated by the local inflammatory milieu. Whether loss of tolerance to gluten in CD patients is caused by such defective Foxp3^+^ cells remains to be established.

## Supporting Information

Figure S1 **Analysis of CD4^+^ CD62L/CD38 T-cell subsets in peripheral blood of adult CD patients and controls.** Peripheral blood was obtained from adult patients with RCD (n = 14), CD patients responding to a GFD (treated CD, n = 15) and healthy controls (n = 14). **(a)** The percentages of total CD4^+^ T cells (within CD3^+^ T-cell gate). **(b)** The percentage of CD45RA^+^ naive T cells within the CD3^+^CD4^+^ T cell gate. **(c)** De percentage of total CCR9^+^ cells within the total CD3^+^CD4^+^ T cell gate or **(d)** within the different CD62L/CD38 T cells. CD, celiac disease; RCD, refractory celiac disease.
(EPS)

Figure S2 **Increased numbers of Foxp3^+^ cells in the lamina propria of pediatric CD patients. (a-d)** Immunohistochemical detection of Foxp3 on paraffin embedded duodenal biopsies from pediatric controls and CD patients. **(e)** Isotype control antibody, mouse IgG_1_, staining. Original magnification:×20. Figures are representative for 16 different CD patients and controls. CD, celiac disease.
(EPS)

Figure S3 **Increased numbers of Foxp3^+^ cells in the lamina propria of adult CD.** **(a-e)** Immunohistochemical detection of Foxp3 on paraffin embedded duodenal biopsies from healthy controls, treated CD and RCD patients. Original magnification:×20. Figures are representative for 12 different patients and controls. CD, celiac disease; RCD, refractory celiac disease.
(EPS)
